# Melatonin enhances plant growth and abiotic stress tolerance in soybean plants

**DOI:** 10.1093/jxb/eru392

**Published:** 2014-10-06

**Authors:** Wei Wei, Qing-Tian Li, Ya-Nan Chu, Russel J. Reiter, Xiao-Min Yu, Dan-Hua Zhu, Wan-Ke Zhang, Biao Ma, Qing Lin, Jin-Song Zhang, Shou-Yi Chen

**Affiliations:** ^1^State Key Laboratory of Plant Genomics, Institute of Genetics and Developmental Biology, Chinese Academy of Sciences, Chaoyang District, Beichen West Road, Campus #1, No.2, Beijing 100101, China; ^2^Beijing Key Laboratory of Genome and Precision Medicine Technologies, The DNA Sequencing Technologies R&D Center, Beijing Institute of Genomics, Chinese Academy of Sciences, Chaoyang District, Beichen West Road, Campus #1, No.7, Beijing 100101, China.; ^3^Department of Cellular and Structural Biology, University of Texas Health Science Center, San Antonio, Texas 78229-3900, USA; ^4^Institute of Crop and Nuclear Technology Utilization, Zhejiang Academy of Agricultural Sciences, Shiqiao Road No.198, Hangzhou City 310021, China

**Keywords:** Melatonin, soybean, yield increase, stress tolerance, transcriptome.

## Abstract

Melatonin is a well-known agent that plays multiple roles in animals. Its possible function in plants is less clear. In the present study, we tested the effect of melatonin (*N*-acetyl-5-methoxytryptamine) on soybean growth and development. Coating seeds with melatonin significantly promoted soybean growth as judged from leaf size and plant height. This enhancement was also observed in soybean production and their fatty acid content. Melatonin increased pod number and seed number, but not 100-seed weight. Melatonin also improved soybean tolerance to salt and drought stresses. Transcriptome analysis revealed that salt stress inhibited expressions of genes related to binding, oxidoreductase activity/process, and secondary metabolic processes. Melatonin up-regulated expressions of the genes inhibited by salt stress, and hence alleviated the inhibitory effects of salt stress on gene expressions. Further detailed analysis of the affected pathways documents that melatonin probably achieved its promotional roles in soybean through enhancement of genes involved in cell division, photosynthesis, carbohydrate metabolism, fatty acid biosynthesis, and ascorbate metabolism. Our results demonstrate that melatonin has significant potential for improvement of soybean growth and seed production. Further study should uncover more about the molecular mechanisms of melatonin’s function in soybeans and other crops.

## Summary

We demonstrate, using the seed-coating method, that melatonin promotes soybean growth, seed production, and stress tolerance by regulating cell division, photosynthesis, carbohydrate metabolism, fatty acid biosynthesis, and ascorbate metabolism.

## Introduction

Extracts of the pineal gland were shown to lighten the skin colour of tadpoles, frogs and fish. In 1958, the active molecule, isolated from bovine pineal glands, was identified as *N*-acetyl-5-methoxy-tryptamine, also known as melatonin (Lerner *et al.*, 1958; Lerner *et al.*, 1960). Melatonin is now a well-known animal hormone that has several important biological functions, including influencing circadian rhythms (Hardeland *et al.*, 2012), mediating changes in seasonal reproduction (Barrett and Bolborea, 2012), immuno-enhancement (Calvo *et al.*, 2013), tumour inhibition (Blask *et al.*, 2005; Bizzarri *et al.*, 2013), and reducing oxidative stress (Hardeland *et al.*, 1993; Reiter *et al.*, 2000; Gitto *et al.*, 2001; Silva *et al.*, 2004; Galano *et al*., 2011, 2013).

In 1995, using HPLC (high performance liquid chromatography) and radioimmunoassay, researchers identified melatonin in plants (Dubbels *et al.*, 1995; Hattori *et al.*, 1995; Van Tassel *et al.*, 1995). Later research revealed that melatonin is also present in unicellular organisms (Hardeland and Poeggeler, 2003).

The biosynthesis of melatonin begins with tryptophan (Reiter, 1991). Vascular plants have similar biosynthetic pathways as that in animals (Arnao and Hernandez-Ruiz, 2006) and homologous enzymes in plants have been identified (Fujiwara *et al.*, 2010). In 2011, the final enzyme in the melatonin biosynthesis pathway was identified in rice as *N*-acetylserotonin methyltransferase (ASMT; Kang *et al.*, 2011), which has a rate-limiting role. Research in rice has also revealed some differences in melatonin synthesis from other organisms; for example, the first metabolite in rice is tryptamine, but not 5-OH Trp (Kang *et al.*, 2007; Park *et al.*, 2012).

Melatonin may possess a variety of functions in vascular plants (Kolar and Machackova, 2005; Uchendu *et al.*, 2013). One of the important roles of melatonin is to act as an antioxidant and protect plants against biotic/abiotic stress (Tan *et al.*, 2012). This antioxidative effect of melatonin has been reported in several plant species (apple, rice, and grape) (Wang *et al.*, 2012; Park *et al.*, 2013; Vitalini *et al.*, 2013; Yin *et al.*, 2013). Using high-throughput sequencing technology, the important roles of melatonin in plant defence have also been revealed. Melatonin up-regulates transcript levels of many defence-related factors, including stress receptors, kinases, and transcription factors (Weeda *et al.*, 2014). Additionally, melatonin may have the ability to regulate plant growth and to enhance crop production. For example, melatonin was reported to promote coleoptile growth in four monocot species including canary grass, wheat, barley, and oat (Hernandez-Ruiz *et al.*, 2005). Melatonin also promotes root growth in *Brassica juncea* (Chen *et al.*, 2009) and adventitious root regeneration in shoot tip explants of sweet cherry (Sarropoulou *et al.*, 2012). Additionally, melatonin-treated corn plants had greater production than non-treated plants (Tan *et al.*, 2012). However, melatonin’s broad functions and its molecular mechanisms in important crops remain unclear.

Soybean is an important crop for oil and as a protein resource. Previous studies have shown that Alfin-like and NAC transcription factors from soybean enhance salt tolerance in transgenic *Arabidopsis* (Wei *et al.*, 2009; Hao *et al.*, 2011) and DOF, bZIP, and MYB transcription factors promote oil accumulation (Wang *et al.*, 2007; Song *et al.*, 2013; Liu *et al.*, 2014). In this study, we investigated the potential roles of melatonin in regulation of soybean growth, yield-related traits, and stress tolerance. We found that melatonin promoted plant growth, increased yield, and improved abiotic stress tolerance. Transcriptome analysis revealed that melatonin may exert its functions mainly through regulation of photosynthesis, the cell cycle, DNA replication, starch/sucrose metabolism, and lipid biosynthesis.

## Materials and methods

### Melatonin application

Melatonin was dissolved in 100% ethanol (EtOH) at a concentration of 30mM and stored at –20 °C. For coating seeds with melatonin, storage solution was diluted to 1mM with 100% EtOH and then further diluted to different concentrations (0 µM, 50 µM, 100 µM) with seed-coating-reagent (Bayer, Germany). Soybean seeds were coated with 300 µl per 100-seed reagent and dried in the air at room temperature. For the RNA-sequencing experiments, storage solution was diluted to 1mM with 100% EtOH and then further diluted to 100 µM with water.

### Growth conditions

The soybean seeds (Glycine max, SuiNong 28, SN28) were sowed in pre-watered soil. The seedlings were grown in a sunlit greenhouse, with the temperature about 25 °C at night and 30–35 °C during the day. The size of the unifoliate and trifoliate was measured during their growth. Agronomic traits, including pods per plant, seeds per plant, and 100-seed weight were calculated. Thirty plants of each concentration were measured and the experiment was repeated independently. A t-test was performed to detect significant differences compared with control plants.

### Performance of soybean plants in field test

Melatonin-coated soybean seeds were sowed in the experimental station of our institute in Beijing (located at 40°22′ N and 116°22′ E). The soil was first watered and then soybean seeds were sowed with a spacing of about 7cm. To ensure the germination rate, three seeds were sowed in one hole. If more than one seedling germinated at each site, only the healthiest seedling was kept and the others were removed within 3 weeks. Thirty plants from each row were measured for agronomic traits after harvest.

### Evaluation of the plants under stress

Melatonin-coated soybean seeds were sowed in greenhouse. For the salt-stress test, seven-day-old seedlings were transferred to soil saturated with 1% (w/v) NaCl. The seedlings were grown at 25 °C under artificial light (about 20,000 LUX) with a photoperiod of 16-h light and 8-h dark. The phenotypes were analysed at one and three weeks later. Thirty six plants of each concentration were measured for plant height and leaf area; ten plants of each concentration were measured for biomass and five plants were measured for EL. For the drought-stress test, seven-day-old seedlings were tested for their performance. The soil used in this experiment was completely crushed and mixed with vermiculite. This mixed soil has the water capacity of 120% (w/w). The water supply was interrupted for about 12 d and the pot weight was measured every 2 d until the water content dropped to 20% of field capacity. The plants were kept under this drought condition for 10 d (with proper water supplement every day if water content was below 20%) and then the plants from above the cotyledon node were harvested. The plants were dried at 75 °C for at least 2 d and then their biomass was measured (dry weight). The value of biomass was compared with the well-watered plants and the reduction in biomass was calculated (Harb and Pereira, 2011). Ten plants of each concentration were measured for biomass. Both salt and drought experiments were repeated independently and a t-test was performed to detect significant differences compared with control plants.

### Chlorophyll content measurement

After treatment in 1% NaCl for 3 weeks, the leaves of soybean were cut for a chlorophyll assay. The fresh weight of leaves was measured (m). The leaves were ground with silica sand and 1ml of 95% EtOH. The mortar was washed with 95% EtOH and all of the EtOH was transferred to clean tubes with a final volume of 25 (V) ml. Chlorophyll was measured with spectra of 645nm and 663nm using spectrophotometer. Chlorophyll A (mg g^–1^)=(12.72A_663_–2.59A_645_)×V/(m×1000), chlorophyll B (mg g^–1^)= (22.88A_663_–4.67A_645_)×V/(m×1000). Three seedlings of each concentration were used in a chlorophyll assay.

### Relative electrolyte leakage assay

After treatment in 1% NaCl for about 3 weeks, the first trifoliate was cut for the relative electrolyte leakage assay. The leaf was vacuumed and placed at room temperature for 2h. Conductivity (K1) was then measured. Bottles containing the leaves were also autoclaved for 15min to completely destroy the leaves. The samples were shaken at 200rpm at room temperature for 1h. Conductivity (K2) was measured again. REL (relative electrolyte leakage) was calculated as K1/K2.

### DAB staining

Five-day-old seedlings were transferred into soil containing 1% (w/v) NaCl and maintained for about 3 weeks. The central-trifoliate was cut and soaked in 1mg ml^–1^ DAB (diaminobenzidine) solution (50mM Tris-HCl pH 4.0). After vacuum infiltration, the soybean leaf became translucent. Following DAB staining for one day and decolouration with absolute alcohol, the brown colour on the leaves indicated presence of hydrogen peroxide.

### RNA extracting and sequencing

Three-week old seedlings were treated with water, 100 µM melatonin, 1% NaCl or 100 µM melatonin plus 1% NaCl. Because gene expression in response to environmental change is a relatively quick process, seed-coating-reagent is not appropriate for this experiment owing to its slow-releasing effect. Therefore, melatonin was directly supplied to soybean seedlings with aqueous solution. Total RNA was extracted using TRNzol Reagent (TIANGEN company). RNA-sequencing was performed by GENEWIZ company using Illumina HiSeq. After cutting off the adaptor sequence and deleting low-quality reads, raw reads were mapped to the soybean genome (http://www.plantgdb.org) using software BWA (Burrows-Wheeler Alignment, bwa-0.7.4). Differentially expressed genes were analysed using the RPKM method (reads per kilo bases per million reads): RPKM=10^9^C/NL. “C” identifies a read number that uniquely mapped to a certain gene. “N” identifies a read number that uniquely mapped to the entire genome. “L” identifies the length of a certain gene. Gene ontology (GO) annotation and enrichment analyses were performed using a Blast2Go and GO-TermFinder (0.86) based on results of blastx. Up-/down-regulated transcripts (fold change ≥2) were examined for common genes using an online Venn diagram tool (http://bioinfogp.cnb.csic.es/tools/venny/index.html). Gene function was then annotated on KAAS (KEGG Automatic Annotation Server). Further detailed analysis was performed using perl program. Quantitative RT-PCR was performed to test the results of RNA-Seq using RAN extracted from independently grown and treated seedlings. The primers of qRT-PCR are found in Supplementary Table S4. Raw data of RNA-Seq was uploaded to NCBI (GEO accession number: GSE57960).

### Fatty acid content analysis

Seeds from a field test were analysed for their fatty acids (FA) content. Soybean seeds were ground to a fine powder and FA were extracted based on a previously published method (Poirier *et al.*, 1999) and analysed by gas chromatography (GC2014, SHIMADZU).

## Results

### Melatonin improves the growth and yield when coated onto soybean seeds

**Fig. 1.**
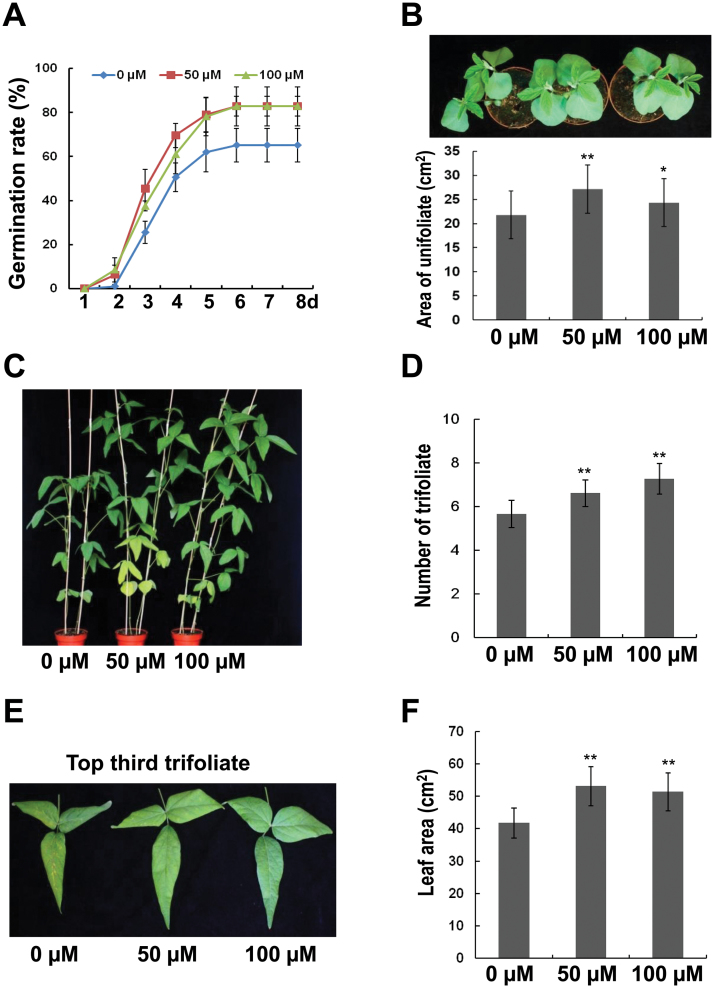
Melatonin effects on soybean growth in a greenhouse using the seed-coating method. (A) Germination rate of soybean seeds coated with different concentrations of melatonin. (B) Melatonin effects on leaf growth. Upper panel: leaf phenotype after treatment. Lower panel: measurement of leaf area. (C) Phenotype of five-week-old soybean seedlings after melatonin treatment. (D) Number of trifoliate after melatonin treatment. (E) The top third trifoliate of 11-week old seedlings after melatonin treatment. (F) Leaf area of central-trifoliate after melatonin treatment. For B, D, and F, * and ** indicate significant difference (*P*<0.05 and *P*<0.01, respectively) compared with mock coating (0 µm). Bars indicate standard deviation (*n*=30).

During agricultural procedures, soybean seeds are usually coated with seed coating-reagent for protection. In the present study we coated soybean seeds with seed-coating-reagent (Bayer, Germany) containing different concentrations of melatonin and sowed them in a greenhouse. Coated seeds were sowed in potted soil with saturated water irrigation and germination rate was assessed every day. A higher concentration (200 µM) of melatonin had no significant effect (Supplementary Fig. S1) or even inhibitory effect (Hernandez-Ruiz *et al.*, 2004) on seed germination. However, lower concentrations of melatonin (50 or 100 µM) promoted seed germination when compared with the control treatment (Fig. 1A). Most seeds germinated between the third to fifth day after sowing and these seedlings were used for further analysis. The seeds that germinated too early or too late were abandoned. Seedlings from melatonin-coated seeds had significantly larger leaves than seedlings from control-coated seeds (0 µM) (Fig. 1B). Because of the slow-releasing effect of coating-reagent, this phenomenon was observed two to three weeks after sowing. In the fifth week, melatonin-treated plants were taller and developed one more trifoliate leaf than the control plants (Fig. 1C, D). Before harvest, the central leaf of the third trifoliate from the top, which was fully expanded, was measured. The trifoliate leaves of melatonin-treated plants were much larger than those of the control seedlings (Fig. 1E, F). These results indicate that melatonin promotes soybean growth and development.

**Fig. 2.**
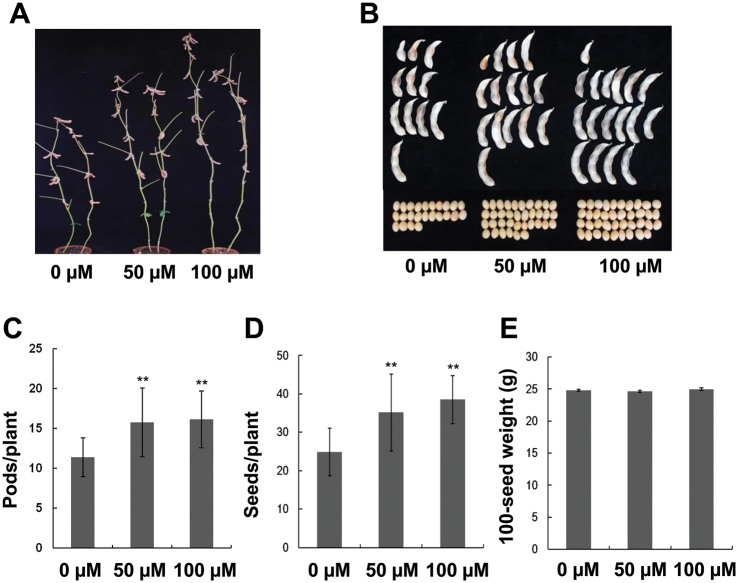
Yield-related traits from soybean plants grown in a greenhouse. (A) Phenotypes of soybean plants before harvest after treatments with different concentrations of melatonin. (B) Pods and seeds from plants with different treatments of melatonin. (C) Comparison of pod numbers after melatonin treatment. (D) Seed numbers in plants treated with melatonin. (E) Weight of 100 seeds after melatonin treatment. For C and D, ** indicate significant difference (*P*<0.01) compared with mock coating (0 µM). Bars indicate standard deviation (*n*=30). (This figure is available in colour at *JXB* online.)

Three months after germination, soybean seeds were harvested and agronomic traits were measured. Melatonin-treated soybean plants produced more pods and seeds than the controls (Fig. 2A–D). However, the 100-seed weight was not significantly influenced (Fig. 2E). These results indicate that melatonin increases yield of soybean plants grown in pots.

### Performance of melatonin-treated soybean plants in a field test

**Fig. 3.**
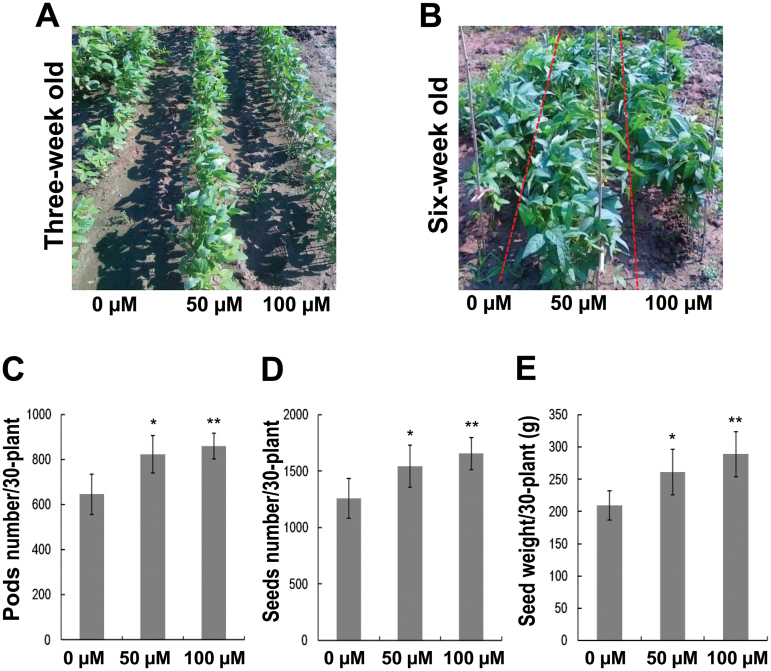
Melatonin effects on soybeans grown under field conditions. (A) Comparison of three-week-old plants grown in the field. (B) Phenotypes of six-week-old plants. (C) Comparison of pod numbers from 30 plants. (D) Seed numbers from 30 plants. (E) Seed weight from 30 plants. For C, D, and E, * and ** indicate significant difference (*P*<0.05 and *P*<0.01 respectively) compared with mock coating (0 µM). Bars indicate standard deviation (*n*=4).

Soybean seeds coated with 0, 50, or 100 µM melatonin were sowed in four different regions of the same field in the experimental station. Melatonin-treated and untreated plants were grown in rows, one close to each other, and each row had roughly 70 holes. Melatonin-treated plants grew bigger than control seedlings (Fig. 3A, B). After harvest, yield-related traits were measured. Melatonin-treated plants produced more pods, more seeds and more yield than control plants (Fig. 3C–E). The results suggest that melatonin improves plant growth and soybean production under field conditions. An independent field test was also performed in Zhejiang Province and consistent enhancement in soybean yield was observed (data not shown).

### Melatonin increases salt and drought tolerance of soybean

**Fig. 4.**
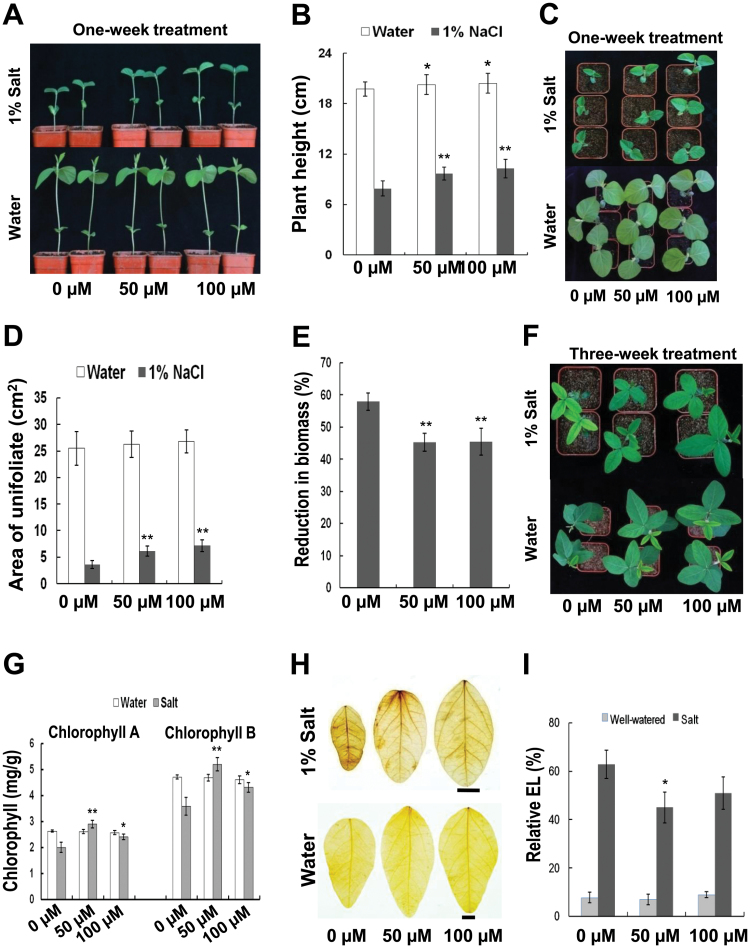
Performance of melatonin-treated seedlings in response to salt stress. (A) Melatonin effects on seedlings treated with 1% salt for one week. (B) Melatonin action on plant height after salt stress. (C) Phenotypes of one-week-old treated seedlings after melatonin and salt treatments. (D) Comparison of leaf area after treatments. (E) Reduction in biomass after treatments. Reduced proportion of biomass (dry weight)=[(biomass of well-watered plants)–(biomass of salt-treated plants)]/(biomass of well-watered plants). (F) Phenotypes of three-week-old treated seedlings. (G) Chlorophyll contents in soybean leaves after salt stress. Left part represents content of chlorophyll A, and right part represents content of chlorophyll B. (H) DAB staining. Brown colour indicates accumulation of H_2_O_2_. Bars=1cm. (I) Relative electrolyte leakage in treated plants. * and ** indicate significant differences (*P*<0.05 and *P*<0.01, respectively) compared with mock coating (0 µM). Bars indicate standard deviation. For leaf area and plant height, *n*=36; for biomass analysis, *n*=10; for chlorophyll test, *n*=3; for relative electrolyte leakage, *n*=5.

We further tested whether melatonin had any effects on abiotic stress responses in soybean plants. Five-day-old seedlings from melatonin-coated seeds were grown in soil with 1% (w/v) NaCl. One week later, leaf area and plant height were measured. Melatonin-treated seedlings were taller and had larger leaves than the control plants (Fig. 4A–D). The treated plants also had a smaller reduction of biomass when compared with the control plants (Fig. 4E). During the third week, the leaves of the control seedlings turned yellow, whereas melatonin-treated seedlings were still green (Fig. 4F). Chlorophyll content was also measured and melatonin-treated plants had similar chlorophyll content as those untreated plants under normal conditions. However, these plants had higher chlorophyll contents than that of control plants after salt treatment (Fig. 4G). DAB staining documented that the control seedlings had higher H_2_O_2_ levels than the melatonin-treated seedlings as the leaves of the control seedlings had a deeper brown colour (Fig. 4H). The relative electrolyte leakage was lower in melatonin-treated seedlings compared with the control seedlings under salt stress (Fig. 4I). These findings imply that melatonin increases salt tolerance in soybean plants.

**Fig. 5.**
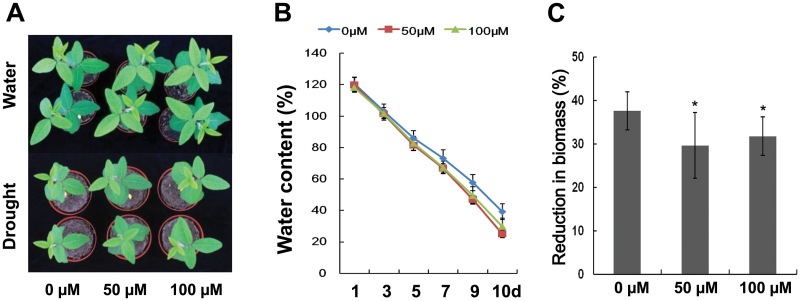
Growth of melatonin-treated seedlings in response to drought stress. (A) Performance of soybean seedlings grown in well-watered soil or in soil supplied with 20% water for one week. (B) Water content of the pot-grown plants after drought stress. (C) Reduction in biomass after drought stress. * indicates significant difference (*P*<0.05) compared with mock coating (0 µM). Bars indicate standard deviation (*n*=10).

One-week old seedlings from melatonin-coated seeds were used to test the drought response of plants, and the water supply was discontinued until the moisture content dropped to 20%. Water content dropped a bit faster in melatonin-treated seedlings than that of control seedlings (Fig. 5B). However, under this condition, melatonin-treated seedlings were larger and had less reduction of biomass compared with controls (Fig. 5A and C). The results suggest that melatonin enhances drought tolerance of soybean plants.

### Melatonin-regulated gene expression by transcriptome analysis

**Fig. 6.**
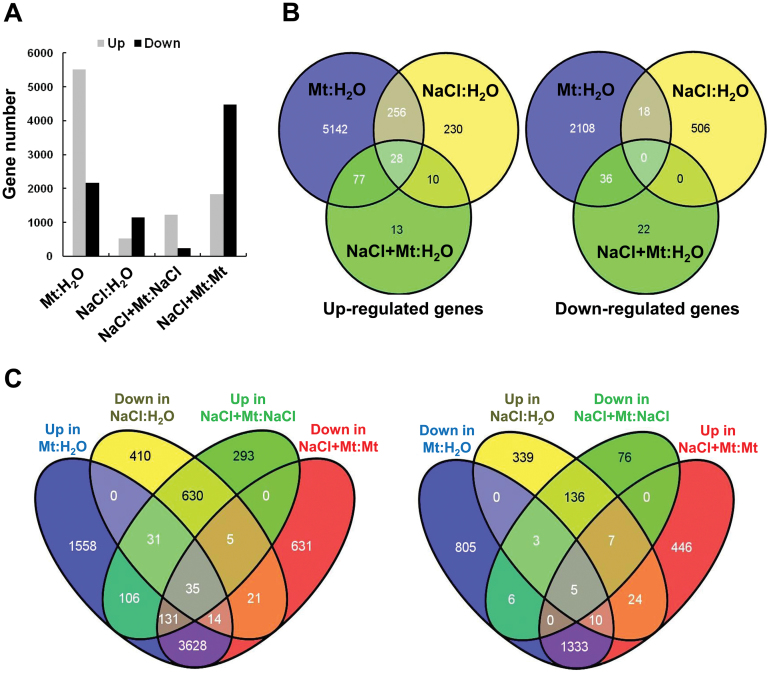
Analysis of RNA-sequencing data. (A) Differentially expressed gene number. Mt:H_2_O identifies 100 µM melatonin-treated samples versus water controls, NaCl:H_2_O identifies 1% salt-treated samples versus water controls, NaCl+Mt:NaCl identifies salt- and melatonin-treated samples versus 1% salt- treated samples, NaCl+Mt:Mt identifies salt- and melatonin-treated samples versus melatonin-treated samples. (B) Gene numbers affected by various treatments. Up- and down-regulated genes (fold change≥2) were examined for common genes using Venn diagram. Overlapping areas represent common genes. NaCl+Mt:H_2_O identifies salt- and melatonin-treated samples versus water controls. (C) Comparison of gene numbers affected by different treatments using Venn diagram.

To investigate the possible mechanism of the promotional roles of melatonin on soybean plants, transcriptome analysis was performed. Two-week old soybean seedlings were treated with water, 100 µM melatonin, 1% NaCl or 1% NaCl plus 100 µM melatonin, and RNAs were isolated for RNA-seq analysis. Statistics of clean reads in RNA sequencing are shown in Table 1. Four comparisons were conducted, including treatments of melatonin (Mt) versus water (Mt:H_2_O), salt versus water (NaCl:H_2_O), salt plus melatonin versus salt (NaCl+Mt:NaCl), and salt plus melatonin versus melatonin (NaCl+Mt:Mt). Compared with the transcripts of non-treated samples (water), melatonin-treated samples had 5503 up-regulated genes and 2162 down-regulated genes, whereas salt-treated samples had 524 up-regulated genes and 1146 down-regulated genes. Compared with salt-treated samples, NaCl+Mt samples had 1231 up-regulated genes and 233 down-regulated genes. Compared with melatonin-treated samples, NaCl+Mt samples had 1825 up-regulated genes and 4465 down-regulated genes (Fig. 6A). The heatmap by cluster analysis also revealed that melatonin enhanced the expression level of a large number of genes compared with the other three samples (Supplementary Fig. S2). Venn diagrams were used to analyse the relationship between different treatments. Compared with water samples, there were 28 genes up-regulated by all three treatments (Fig. 6B and Supplementary Table S1), suggesting that they may respond to environmental changes. It was presumed from the experiments above that melatonin may mitigate the effects of salt (Fig. 4 and Fig. 6A), and thus the regulation of gene expressions by melatonin and salt were analysed. There were 303 (Fig 6C, left: up in Mt:H_2_O versus up in NaCl+Mt:NaCl) genes commonly up-regulated and 14 (Fig 6C, right: down in Mt:H_2_O versus down in NaCl+Mt:NaCl) genes commonly down-regulated by melatonin in the absence and presence of salt. There were 75 (Fig 6C, left: down in NaCl:H_2_O versus down in NaCl+Mt:Mt) genes commonly down-regulated and 46 (Fig 6C, right: up in NaCl:H_2_O versus NaCl+Mt:Mt) genes commonly up-regulated by salt in the absence and presence of melatonin. Four comparisons could be divided into two groups, and each group contained two contrasting comparisons (Group I: Mt:H_2_O and NaCl+Mt:Mt, Group II: NaCl:H_2_O and NaCl+Mt:NaCl) (Fig. 6C). A reciprocal analysis was also performed and much fewer common genes were found (Supplementary Fig. S3). Details of the genes in the Venn diagrams (Fig. 6C) can be found in Supplementary Table S2.

**Table 1.** Statistics of clean reads in RNA sequencing

Samples	Length	Total reads	Total mapped	Unique mapped	Mapped (%)	Unique mapped (%)	Seq depth
Melatonin	100	39 128 572	29 090 309	24 017 624	74.35	82.56	28.7
H_2_O	100	39 614 632	29 231 294	23 910 361	73.79	81.80	29.0
NaCl	100	49 078 288	36 222 072	29 620 331	73.80	81.77	35.9
NaCl+Mt	100	63 169 248	46 886 146	38 472 421	74.22	82.05	46.3

**Fig. 7.**
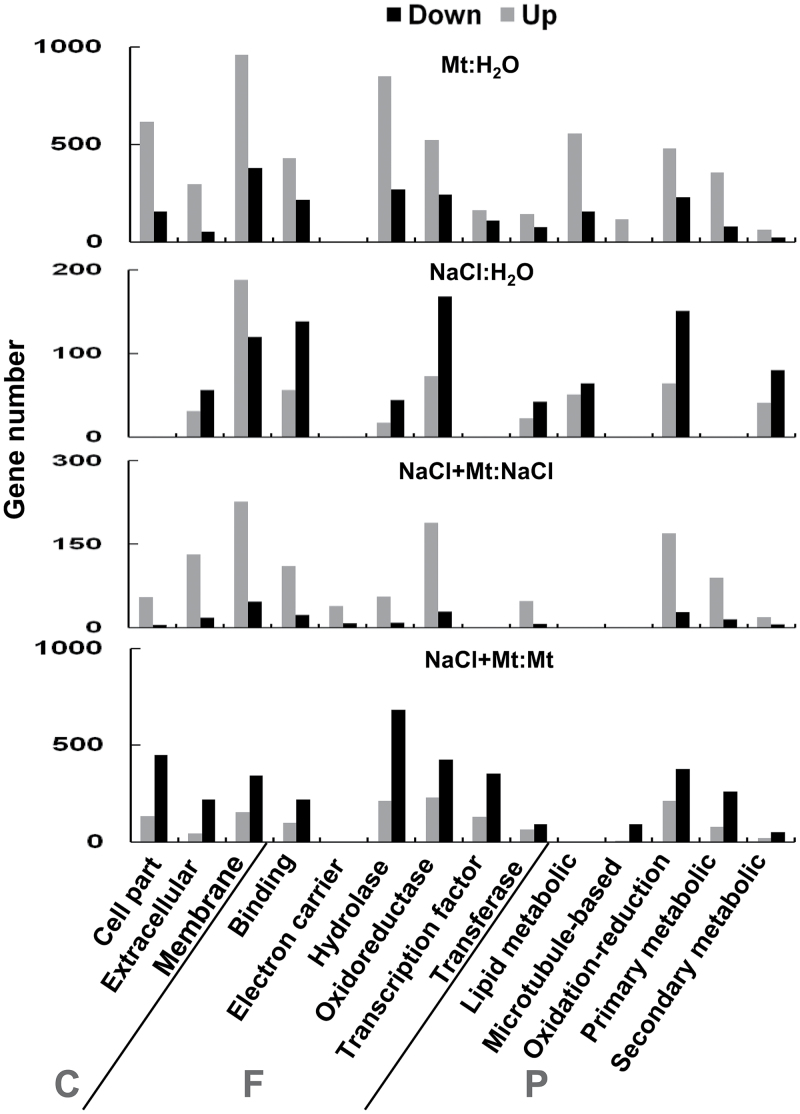
Gene ontology analysis in response to different treatments. C, cellular components; F, molecular functions; P, biological processes.

Gene ontology analysis also was performed (http://www.geneontology.org/). Melatonin exhibited similar regulatory roles in both “Mt:H_2_O” and “NaCl+Mt:NaCl” comparisons, whereas salt stress inhibited related gene expressions. Regulated gene numbers were higher in the “Mt:H_2_O” comparison. Melatonin apparently increased genes related to hydrolase, oxidoreductase, primary metabolism, oxidation-reduction processes, and lipid metabolic processes, whereas salt stress had an inhibitory effect on these processes (Fig. 7).

**Fig. 8.**
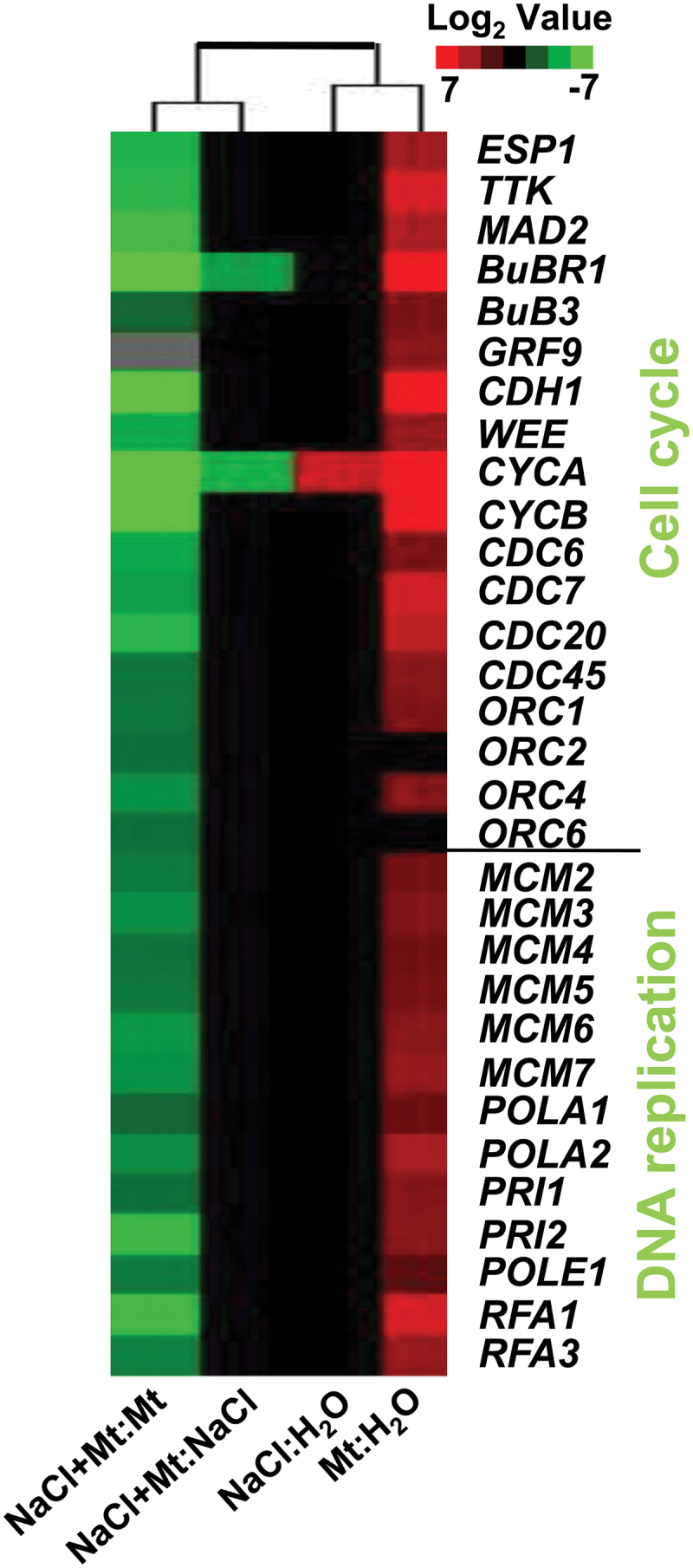
Fold changes of gene expressions in cell cycle and DNA replication processes in three comparisons. The log_2_ (fold change of gene transcripts) value was analysed using cluster software. The annotation of the genes can be found in Supplementary Table S3.

Under non-stress conditions, application of melatonin increased expression level of genes connected to cell cycle and DNA replication processes, including *BUBR1*, *CDH1*, *CYCA*, and *CYCB* genes. However, there was no significant change in gene expressions under salt treatment (Fig. 8, Supplementary Table S3).

**Fig. 9.**
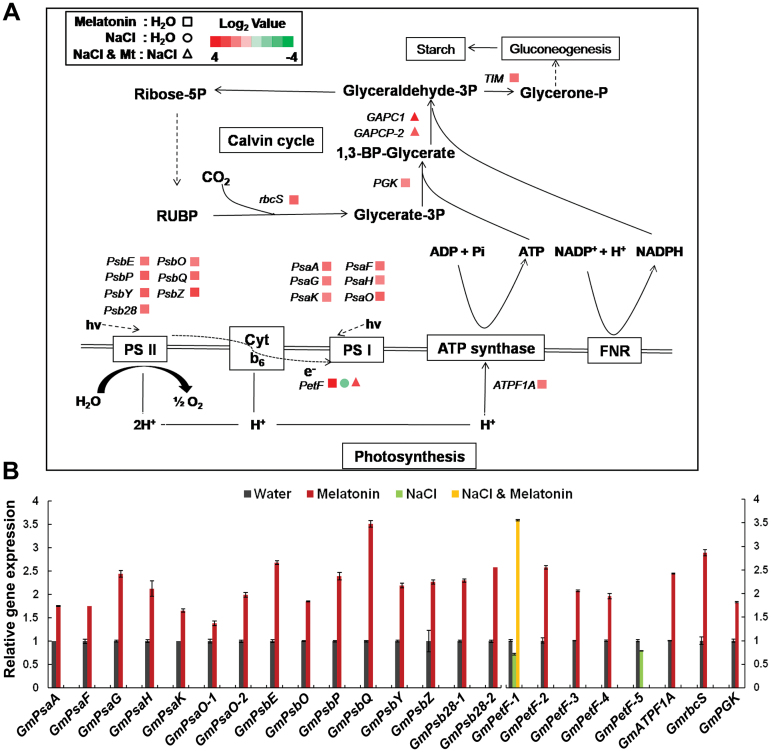
Melatonin enhances expressions of photosynthesis-related genes under normal and salt stress conditions. (A) Expression of genes related to photosynthesis. Red colour indicates up-regulation and green colour indicates down-regulation. Quadrangle represents a comparison of melatonin treated versus water control; circle represents a comparison of salt versus water control: triangle represents a comparison of salt plus melatonin versus salt. (B) Relative gene expression level analysed by q-PCR. A *GmTubulin* fragment was amplified as an internal control. Bars indicate standard deviation (*n*=3).

**Fig. 10.**
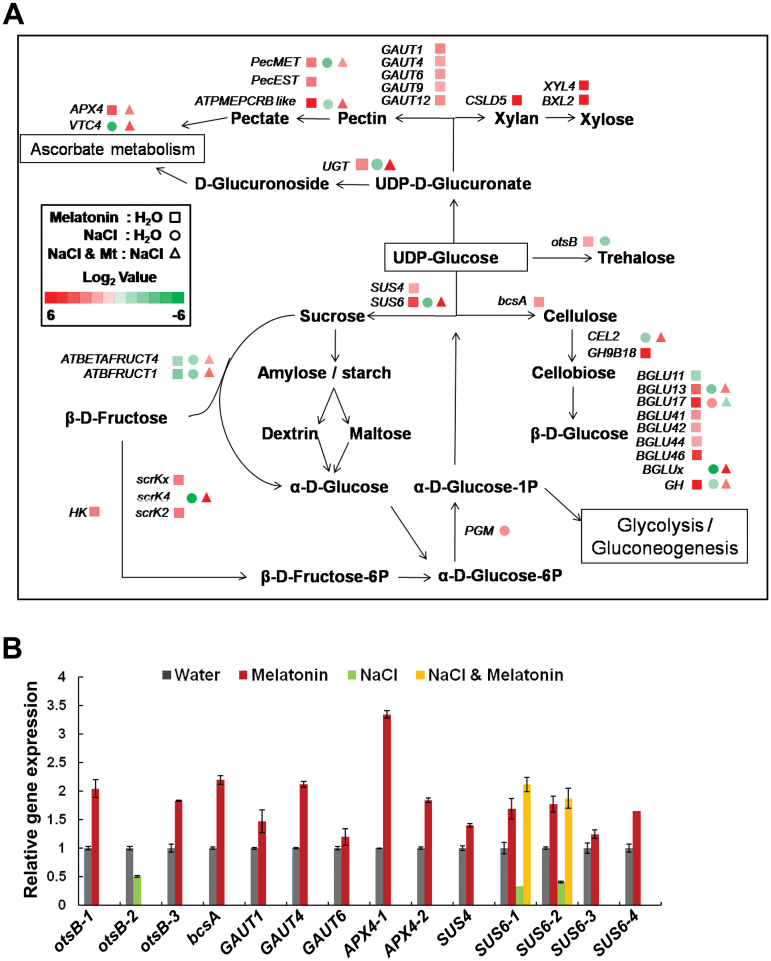
Changes of expression of genes involved in glucose metabolism. (A) Fold changes of gene expressions in the glucose metabolic pathway. (B) Quantitative PCR analysis of gene expression in glucose metabolism. The annotation of the genes can be found in Supplementary Table S3.

**Fig. 11.**
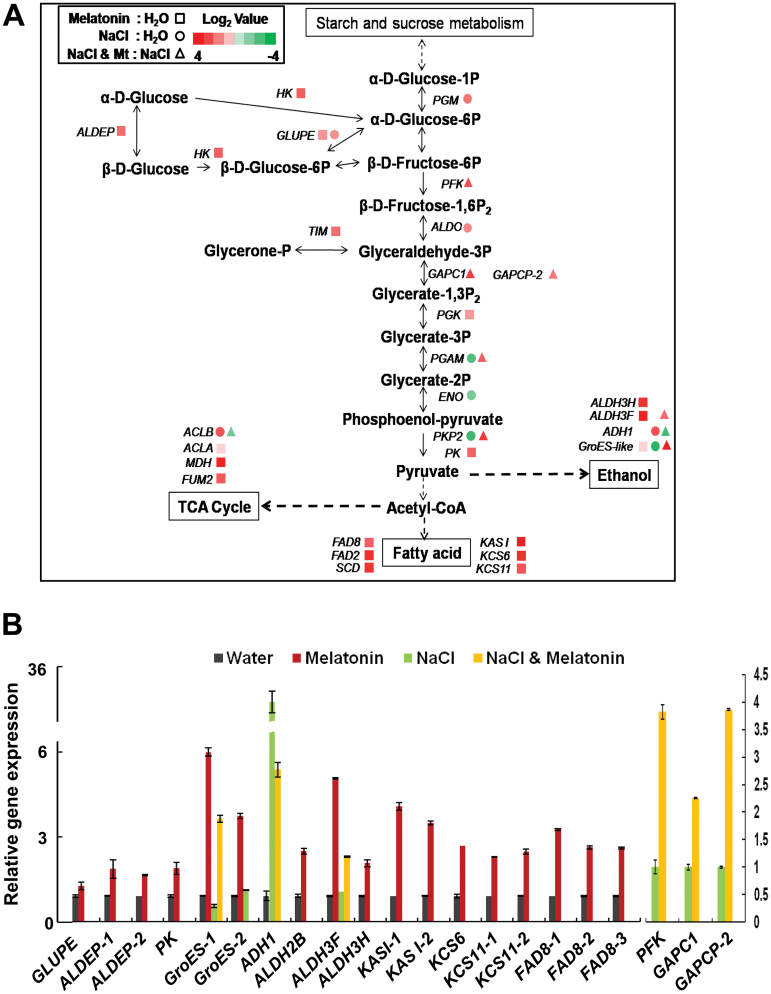
Altered expressions of genes involved in glycolysis, TCA cycle, ethanol metabolism, and fatty acid biosynthesis. (A) Fold change of the expression of genes in the four pathways. Dashed lines indicate omitted steps. (B) Quantitative PCR analysis of genes in glycolysis, ethanol metabolism, and fatty acid biosynthesis.

To confirm the results of transcriptome analysis, we extracted RNA from independently grown and treated plants and performed quantitative RT-PCR. The important genes that enriched in pathway analysis were tested and the real-time PCR results were consistent with transcriptome analysis (Figs 9B, 10B, 11B, and Supplementary Fig. S4B).

### Melatonin up-regulates gene expressions in photosynthesis

Both the photosynthetic light reaction and dark reaction processes were up-regulated by melatonin (Fig. 9A, B). The genes, *PsaA*, *PsaF*, *PsaG*, *PsaH*, *PsaK*, and *PsaO* in photosystem I, and *PsbE*, *PsbO*, *PsbP*, *PsbQ*, *PsbY*, *PsbZ*, and *Psb28* in photosystem II were up-regulated in melatonin-treated plants compared with those in non-treated plants (Fig. 9A, B). Electron transporter genes, *PetF* family, and an F-type ATPase gene *ATPF1A* were also up-regulated in the “Mt: H_2_O” comparison. The *PetF-1* gene was down-regulated in salt-treated plants but this was reversed by melatonin application during salt stress treatment (Fig. 9A, B). In the Calvin cycle, *rbcS*, *GAPC1*, and *GAPCP-2*, which encoded glyceraldehyde-3-phosphate dehydrogenase, were up-regulated by melatonin under normal and salt stress conditions (Fig. 9A). These results show that melatonin improves photosynthesis-related processes under normal and salt stress conditions.

### Gene expression changes in starch and sucrose metabolism

The synthesis genes for sucrose and trehalose were up-regulated by melatonin but down-regulated by salt treatment. Melatonin also activated both the synthesis and degradation of cellulose, pectin, and xylan, whereas salt inhibited these processes for the first two components (Fig. 10A, B). Genes related to ascorbate synthesis and metabolism, including the UDP-glucuronosidase gene, *VTC4*, and *APX4* were also up-regulated by melatonin (Fig. 10A). Melatonin also enhanced some of the above gene expressions during salt stress (Fig. 10A, B).

### Gene expression changes in glycolysis and downstream processes

Under non-stress conditions, gene expression of enzymes that catalyse reactions from glucose to fructose-6P, including *HK*, *ALDEP* and *GLUPE,* were increased by melatonin. The genes *PGK* and *PK* connected with pyruvate biosynthesis were also up-regulated by melatonin. When melatonin was combined with salt treatment, *PFK*, *GAPC1*, *GAPCP-2*, *PGAM*, and *PKP2* were up-regulated. The downstream processes for pyruvate metabolism were also changed by melatonin and salt. For ethanol synthesis and metabolic processes, *GroES-like* genes and *ALDH3* were up-regulated by melatonin. *ADH1* and *GroES-like* genes showed the opposite expression in “NaCl+Mt:NaCl” comparisons. Pyruvate can be catalysed to acetyl-CoA, which further participates in the tricarboxylic acid (TCA) cycle and fatty acid biosynthesis. In the TCA cycle, *ACLA*, *MDH*, and *FUM2* were up-regulated by melatonin. In fatty acid biosynthesis, the *KAS I* and *KCS* gene family were up-regulated by melatonin (Fig. 11A, B). Some of the gene expressions were also further confirmed by quantitative PCR (Fig. 11B). Thus, melatonin promotes glycolysis and facilitates processes involving pyruvate and acetyl-CoA.

**Fig. 12.**
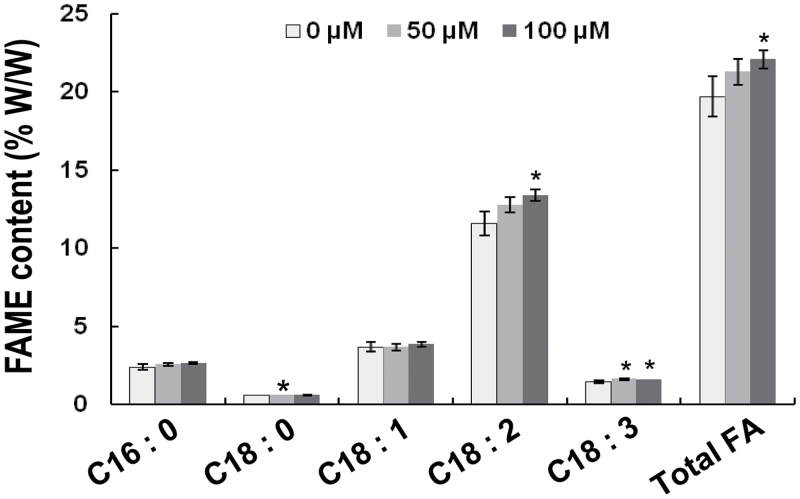
Fatty acid analysis in melatonin-treated plants. Seeds from field-grown plants were analysed for their FA content (% w/w). Bars indicate standard deviation (*n*=3). * indicates significant difference (*P*<0.05) compared with mock coating (0 µM).

As acetyl-CoA is the substrate of *de novo* fatty acid biosynthesis, we further measured fatty acid content in soybean seeds from field-grown plants using gas chromatography. Total FA contents were increased by 1.58% and 2.37% with 50 and 100 µM melatonin treatment, respectively (Fig. 12). These increases were probably due to the major rises of C18:2 composition (Fig. 12).

## Discussion

We have examined the effects of melatonin on soybean plants and found that melatonin, when coated onto seeds, promotes plant growth, development, and yield. It also improved salt and drought stress tolerance. These roles are most likely achieved through enhancement of processes involved in photosynthesis and sugar metabolism. These results provide a novel approach for improving yield of soybeans and possibly of other crops commonly used in agriculture.

Previous studies reported that melatonin enhances root growth in other plants (Arnao and Hernandez-Ruiz, 2007; Chen *et al.*, 2009; Sarropoulou *et al.*, 2012). The present study proved that melatonin also improves soybean growth at both the vegetative stage (Fig. 1) and the reproductive stage (Fig. 2). It also increases abiotic stress tolerance (Figs 4 and 5) and the accumulation of fatty acids in soybean (Fig. 12).

Melatonin has functions in plants that differ from those in animals; one of these is growth improvement (Tan *et al.*, 2012). Melatonin not only enhanced the size of soybean seedlings, but also improved their growth rate (Fig. 1). New trifoliates developed faster when treated with melatonin (Fig. 1C, D). Moreover, melatonin also increased yield of soybean both in greenhouse and in the field (Fig. 2 and 3), suggesting its potential application in agriculture. Like plant hormones, melatonin displayed weak effects at higher concentrations (Supplementary Fig. S1), or even had inhibitory actions (Hernandez-Ruiz *et al.*, 2004).

In the field test, 50 µM melatonin-treated seedlings seemed to be heathier than control seedlings or 100 µM-treated seedlings (Fig. 3A, B). However, 100 µM melatonin-treated plants had much higher seed number than control plants and had slightly higher seed number than 50 µM melatonin-treated plants (Fig. 3D). This fact indicates that high concentrations of melatonin may allow the effects to persist for a long period, thereby more significantly enhancing the yield.

Melatonin improves salt and drought tolerance in soybean plants as observed from the increased height and leaf area, and less biomass reduction when subjected to these stresses (Figs 4, 5). These effects are probably a result of the increased antioxidative ability and more stable membrane systems, as judged from the DAB staining and electrolyte leakage (Fig. 4H, I).

Transcriptome analysis was performed to investigate the possible mechanisms by which melatonin promotes plant growth and stress tolerance. From the observed results, we propose that melatonin may act as an activator of many genes. In the current study, salt stress suppressed many genes, whereas melatonin by yet undefined mechanisms was able to overcome the inhibitory effects of salt stress and reactivated many of the suppressed genes (Fig. 6A and Supplementary Fig. S2). Gene ontology analysis also showed that melatonin promoted the expression of many genes and inhibited the effects of salt stress (Fig. 7). The promotional effects of melatonin on plant growth may be achieved through activation of DNA replication and cell division as many related genes are up-regulated (Fig. 8).

In vascular plants, the photosystem consists of two parts, photosystem I and photosystem II. Two subunits (PsaK and PsaG) in photosystem I can influence plant size because the deletion mutants of them, *psak-1* and *psag-1.4*, had smaller plant size (Varotto *et al.*, 2002). Melatonin enhanced expression levels of *PsaK* and *PsaG* (Fig. 9A), which may further enhance plant size of soybean plants. In photosystem II, water is converted to oxygen and protons in a cluster of oxygen-evolving complexes (OEC) (Cady *et al.*, 2008). PsbO (oxygen-evolving enhancer protein 1/OEE1) is essential for the stabilization of the cluster; and PsbP (OEE2) is required for the oxygen-evolving activity (Mayfield *et al.*, 1987). The expression levels of *PsbO* and *PsbP* may influence the activity of OEC and thus influence plant growth. It has been found that mutation of the *PsbO* gene caused growth retardation in *Arabidopsis* (Murakami *et al.*, 2005). Melatonin up-regulated expression levels of *PsbO* and *PsbP* (Fig. 9A), hence leading to the larger size of soybean plants.

Melatonin enhances ferredoxin gene *PetF* and suppresses salt inhibition of this gene (Fig. 9). Ferredoxin regulates the amount of reduced ascorbate and protects chlorophyll from degradation (Lin *et al.*, 2013). Low expression of *PetF* may affect the scavenging of reactive oxygen species (ROS) generated during photosynthesis or as a result of salt stress, consistent with the growth retardation and H_2_O_2_ accumulation in salt stress (Fig. 4). Melatonin may promote *PetF* expression under salt stress and, hence, reduce H_2_O_2_ accumulation (Fig. 4H and 9). We also found that genes involved in ascorbate metabolism, including *VTC4* and *APX4*, are up-regulated by melatonin under normal and/or salt stress conditions (Fig. 10A). The former gene is involved in biosynthesis of ascorbate (Torabinejad *et al.*, 2009), and the latter gene functions in reducing H_2_O_2_ levels (Panchuk *et al.*, 2005). These findings indicate that melatonin probably also has a role in the promotion of the antioxidative capacity of soybeans.

Sugar metabolism-related genes were also altered by melatonin. Both the synthesis- and degradation-related genes were up-regulated in the melatonin-treated plant, suggesting an active metabolism of sugars possibly for the activated cell division/cell cycle process during enhanced plant growth. These results agree well with GO analysis that primary metabolism was enhanced by melatonin (Fig. 7). Melatonin also promoted the expression of the trehalose synthesis gene. Trehalose is an important carbohydrate that helps plants preserve their cellular integrity under various stresses (Jain and Roy, 2009).

Melatonin enhanced the genes involved in glycolysis under both normal and salt stress conditions (unidirectional arrows in Fig. 11A). Glycolysis is responsible for glucose conversion into pyruvate, which is further converted into acetyl-CoA required for the biosynthesis of fatty acids (Jeoung *et al.*, 2014). Additionally, melatonin raised the expression of a number of genes in fatty acid biosynthesis (Okuley *et al.*, 1994; Millar and Kunst, 1997; Wu and Xue, 2010) (Fig. 11), which accounted for the fatty acid accumulation in soybean seeds (Fig. 12). Recently, we identified a transcription factor, GmbZIP172, which binds and activates the expression of two sucrose transporter genes and three cell-wall invertase genes. In *GmbZIIP172*-overexpressing *Arabidopsis* plants, sucrose and glucose contents are increased in young seeds, leading to elevated level of oil accumulation in mature seeds of transgenic plants (Song *et al.*, 2013).

Gene expression related to changes in amino acid metabolism were also detected (Supplementary Fig. S4A). Tryptophan is the precursor of melatonin and ASMT is the last enzyme of melatonin biosynthesis (Kang *et al.*, 2011). The up-regulation of *ASMT* by exogenous melatonin application suggests the possibility of a positive feedback control of melatonin synthesis (Supplementary Fig. S4B). This mechanism may be the basis for the observation that low amounts of melatonin induced huge and long-lasting promotional effects on plant growth.

The results show that melatonin increases plant growth, seed production, and abiotic stress tolerance in soybean plants, possibly through enhancement of photosynthesis, carbohydrate metabolism, and antioxidative actions. This agent may have great potential for improving crop yield. Further study should examine the molecular mechanisms of melatonin’s functions in plants.

## Supplementary data

Supplementary data are available at *JXB* online

Figure S1. Germination rate of soybean seeds coated with different concentrations of melatonin.

Figure S2. Cluster analysis of the four samples.

Figure S3. Venn diagram analysis of the four comparisons.

Figure S4. Pathways for biosynthesis and metabolism of amino acids.

Table S1. Common genes up-regulated in all treatments compared with H_2_O samples.

Table S2. Details of Venn diagram in Fig. 6C.

Table S3. Gene annotation.

Table S4. Realtime PCR primers.

Supplementary Data
