# Supplementary material for: Melatonin enhances plant growth and abiotic stress tolerance in soybean plants
Source: J Exp Bot. 2014 Oct 6;66(3):695–707. doi: 10.1093/jxb/eru392 (PMC4321538; doi:10.1093/jxb/eru392)
Supplement: Supplementary Data [file supp_66_3_695__index.html]

Melatonin enhances plant growth and abiotic stress tolerance in soybean plants — Melatonin enhances plant growth and abiotic stress tolerance in soybean plants — Supplementary Data 

# Melatonin enhances plant growth and abiotic stress tolerance in soybean plants

## Supplementary Data

Data files

**Files in this Data Supplement:**

- Supplementary Data - Supplementary Data
- Supplementary Data - Supplementary Data
