# Supplementary material for: Melatonin enhances plant growth and abiotic stress tolerance in soybean plants
Source: J Exp Bot. 2014 Oct 6;66(3):695–707. doi: 10.1093/jxb/eru392 (PMC4321538; doi:10.1093/jxb/eru392)
Supplement: Supplementary Data [file supp_eru392_Sup._figure_edited.pdf]

# Melatonin enhances plant growth and abiotic stress tolerance in soybean plants

**Wei Wei[a], Qing-Tian Li[a], Ya-Nan Chu[b], Russel J. Reiter[c], Xiao-Min Yu[d], Dan-Hua Zhu[d], Xin-Lei Liu[e], Wei-Qun Man[e], Wan-Ke Zhang[a], Biao Ma[a], Qing Lin[a], Jin-Song Zhang[a,] *, Shou-Yi Chen[a,] ***

[a]State Key Laboratory of Plant Genomics, Institute of Genetics and Developmental Biology, Chinese Academy of Sciences, Chaoyang District, Beichen West Road, Campus #1, No.2, Beijing 100101, China

[b]Beijing Key Laboratory of Genome and Precision Medicine Technologies, The DNA Sequencing Technologies R&D Center, Beijing Institute of Genomics, Chinese Academy of Sciences, Chaoyang District, Beichen West Road, Campus #1, No.7, Beijing, 100101, China.

[c]Department of Cellular and Structural Biology, University of Texas Health Science Center, 7703 Floyd Curl Drive, MC 7762, San Antonio, TX 78229-3900, USA

[d]Institute of Crop and Nuclear Technology Utilization, Zhejiang Academy of Agricultural Sciences , Shiqiao Road No.198, Hangzhou City 310021, China

[e]Soybean Research Institute, Heilongjiang Academy of Agricultural Sciences, Xuefu Road No.368, Harbin City 150086, China

*Corresponding author

# Sup. Fig. 1

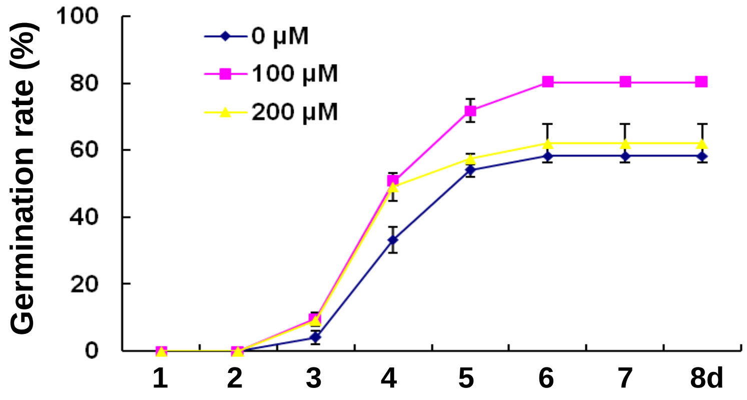


**Sup. Figure 1** Germination rate of soybean seeds coated with different concentrations of melatonin.

## Sup. Fig. 2

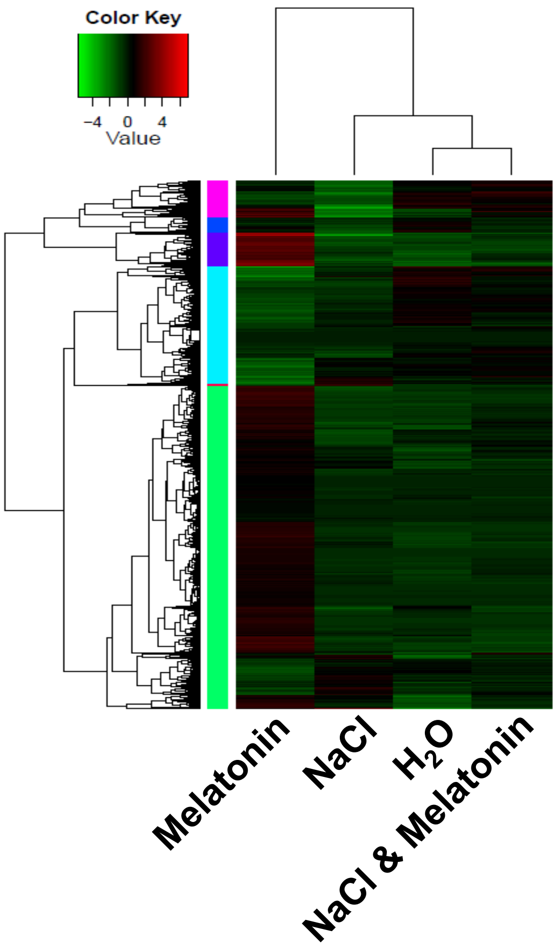


**Sup. Figure 2** Cluster analysis of the four samples. $Log_2$ value = $Log_2$ (Fold change of gene transcripts).

# Sup. Fig. 3

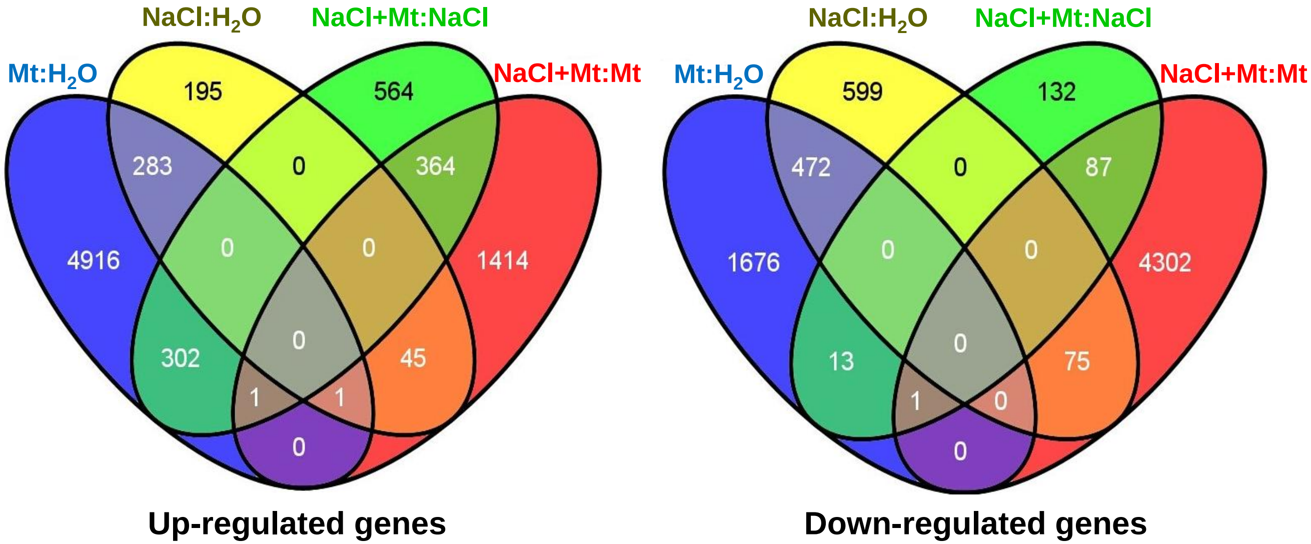


**Sup. Figure 3** Venn diagram analysis of the four comparisons.

Figure on the left demonstrates commonly up-regulated genes, while figure on the right demonstrates commonly down-regulated genes. Overlapping areas represent common genes between different comparisons.

Sup. Fig. 4

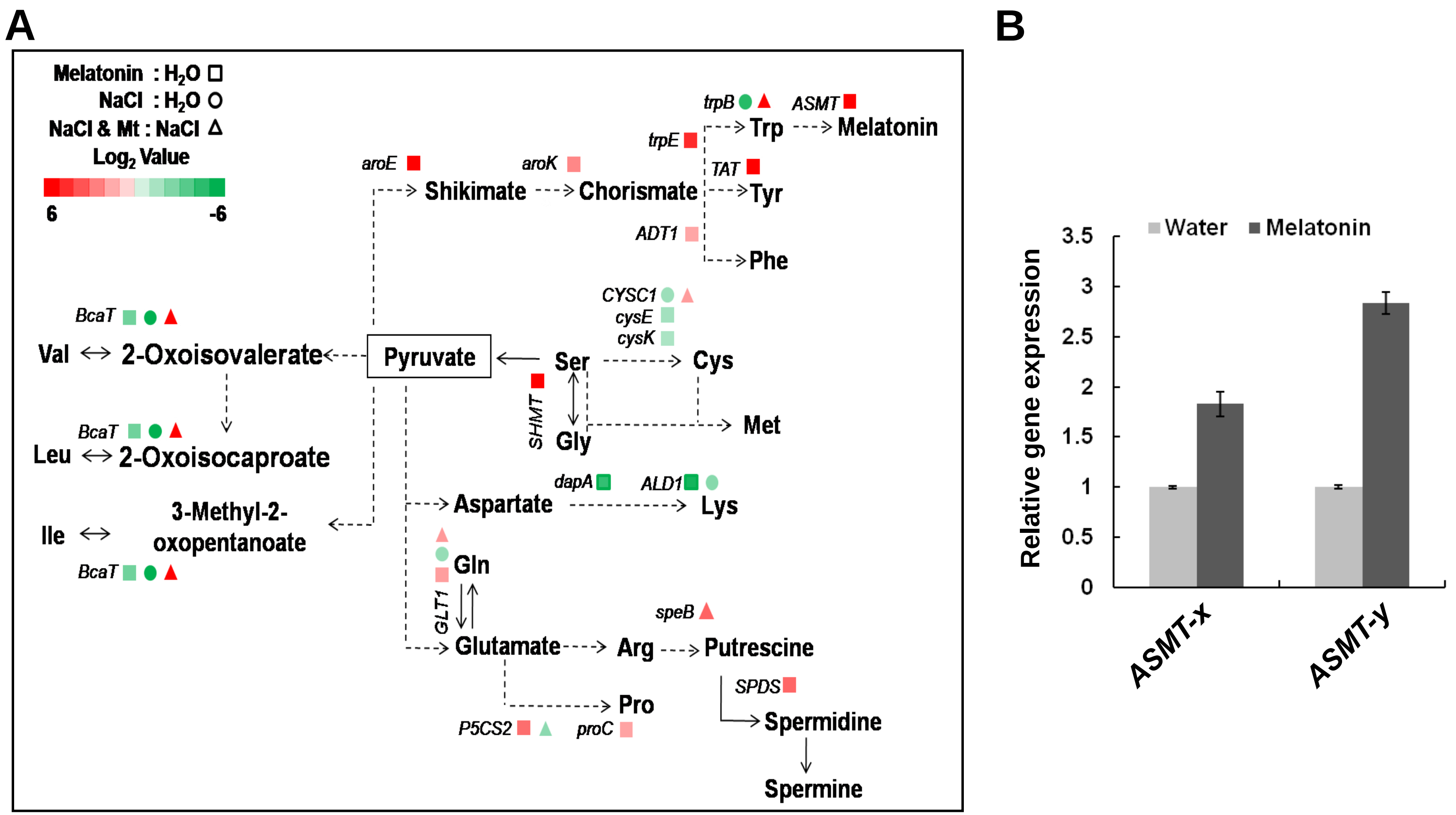


**Sup. Figure 4** Pathways for biosynthesis and metabolism of amino acids. **(A)** Fold change of genes in the pathways. Dashed lines indicate steps omission. **(B)** Quantitative RT-PCR analysis of melatonin synthesis genes.

**Sup. Table 1** Common genes that up-regulated in all treatments compared to $H_2O$ samples.

| Genes | Annotation |
|---|---|
| Glyma13g38050.1 | lyase/ magnesium ion binding |
| Glyma08g01060.1 | CHLM (magnesium-protoporphyrin IX methyltransferase) |
| Glyma03g24450.1 | catalytic/ iron ion binding / oxidoreductase |
| Glyma14g36860.1 | copper-binding family protein |
| Glyma04g35130.1 | RD22; nutrient reservoir |
| Glyma18g41320.1 | protease inhibitor/seed storage/lipid transfer protein (LTP) family protein |
| Glyma07g16770.1 | protease inhibitor/seed storage/lipid transfer protein (LTP) family protein |
| Glyma07g03920.1 | LOX1; lipoxygenase |
| Glyma15g12490.1 | ATMAP70-5 (microtubule-associated proteins 70-5); microtubule binding |
| Glyma18g42520.1 | IRX12 (IRREGULAR XYLEM 12); laccase |
| Glyma20g33450.1 | CAS1 (cycloartenol synthase 1); cycloartenol synthase |
| Glyma18g11820.1 | CYP83B1 (CYTOCHROME P450 MONOOXYGENASE 83B1); oxidoreductase |
| Glyma16g26070.1 | SCPL27 (serine carboxypeptidase-like 27); serine-type carboxypeptidase |
| Glyma03g02130.1 | SBTI1.1; serine-type endopeptidase |
| Glyma04g11370.1 | SEC14 cytosolic factor family protein / phosphoglyceride transfer family protein |
| Glyma16g04760.1 | ATAPY2 (ARABIDOPSIS THALIANA APYRASE 2); ATPase/ nucleotide diphosphatase |
| Glyma06g41520.1 | dihydroflavonol 4-reductase family / dihydrokaempferol 4-reductase family |
| Glyma07g01730.1 | acid phosphatase, putative |
| Glyma11g35130.1 | LHCB4.3 (light harvesting complex PSII); chlorophyll binding |
| Glyma06g05820.1 | DNAJ heat shock N-terminal domain-containing protein |
| Glyma03g24320.1 | CER1 protein, putative |
| Glyma01g39810.1 | AGP17 (ARABINOGALACTAN PROTEIN 17) |
| Glyma11g05470.1 | AGP18 (ARABINOGALACTAN PROTEIN 18) |
| Glyma05g36000.1 | unknown protein |
| Glyma07g01680.2 | unknown protein |
| Glyma07g03920.2 | unknown protein |
| Glyma11g05470.2 | unknown protein |
| Glyma19g01090.2 | unknown protein |
